# The expanding role of biocompatible hydrogels in plant-derived exosome-like nanovesicles for skin diseases: prospects and challenges

**DOI:** 10.1080/07853890.2026.2643038

**Published:** 2026-03-21

**Authors:** Sha Lv, Danyang Fan, Zhanhan Tang, Zhe Liu

**Affiliations:** Department of Dermatology, the Second Hospital of Jilin University, Changchun, China

**Keywords:** Plant exosome-like nanovesicles (PDELNs), exosomes, hydrogels, biomaterials, dermatology, nanomedicine, skincare

## Abstract

**Background:**

Plant-derived exosome like nanovesicles known for their biocompatibility, minimal immunogenicity, and capacity to transport diverse therapeutic molecules, have emerged as effective carriers for targeted drug delivery. When integrated into hydrogels, these offer improved stability, prolonged release, and precise delivery to specific skin layers, thereby enhancing treatment outcomes.

**Methods:**

We conducted structured search of the literature on plant-derived exosome-like nanovesicles (PDELNs) and hydrogel-based drug-delivery systems for skin applications. Relevant studies were identified from PubMed, ScienceDirect, and Google Scholar using keywords including plant-derived exosomes like nanovesicles, plant exosomes + skin, and plant exosomes + hydrogel. We selected Publications from 2010 to 2025 and focused on studies describing biological activity of plant-derived exosomes, as well as their therapeutic relevance for skin repair, regeneration, or wound healing. Research involving hydrogel formulations that incorporated plant-derived vesicles or comparable nano-carriers was also included. *In vitro*, *in vivo* (preclinical), and available clinical evidence were reviewed, while unrelated work (such as mammalian exosomes or non-skin studies) was excluded.

**Results:**

The combination of plant-derived exosomes like nanovesicles and hydrogels is potential therapeutic candidate in addressing various skin disorders, including inflammatory diseases, wound healing, and skin regeneration. However, challenges remain, including the scalability of exosome production, stability of formulations, and achieving effective skin penetration. Furthermore, regulatory considerations regarding the safety, toxicity, and long-term biocompatibility of these systems must be thoroughly evaluated. The review also emphasizes future research opportunities, the development of plant-derived exosomes like nanovesicles with higher therapeutic potential and the integration of advanced technologies like phototherapy and microneedles to further improve therapeutic efficacy.

**Conclusion:**

The combination of PDELNs with hydrogel delivery systems have shown potential in preclinical models for the treatment of dermatological diseases, providing a novel strategy for skin care.

## 1. Introduction

Exosomes are extracellular vesicles, ranges 30 to 150 nanometres in size, and serve as mediators of intercellular communication [1]. Exosomes facilitate the transfer of diverse bioactive molecules, including proteins, lipids, and various forms of RNA. Secreted by nearly all cell types, these vesicles are involved in a wide range of physiological and pathological processes like immune regulation, maintenance of tissue homeostasis, and progression of various diseases [2]. Exosomes facilitate intercellular communication across both short and long distances by delivering their cargo to recipient cells, thereby modulating cellular activities gene expression, protein synthesis, and overall cell behaviour [3]. Exosome biogenesis begins with inward budding of late endosomal membrane, leading to the formation of multivesicular bodies (MVBs) which enclose small intraluminal vesicles (ILVs). Subsequently, these MVBs fuse with the plasma membrane, releasing ILVs into extracellular environment as exosomes [4]. This process is mainly governed by endosomal sorting complex required for transport (ESCRT) machinery, which is crucial for sorting cargo into targeted vesicles for secretion [5]. Lipids and sphingolipids also contribute to the membrane curvature essential for vesicle formation. While the ESCRT pathway is central to exosome biogenesis, alternative mechanisms involving lipid rafts and tetraspanins like CD9, CD63, and CD81 which are recognized as important factors in exosome release [6,7].

After their release into extracellular environment, exosomes specifically target recipient cells. This specificity is mediated by surface proteins tetraspanins, integrins, and lectins, which recognize and bind to complementary molecules on the target cell surface [8]. Through these interactions, exosomes deliver by modulating key cellular processes such as immune responses, cell proliferation, and differentiation [9]. Selective targeting of exosomes ensures precise delivery of their cargo to appropriate cells, enabling controlled modulation of cellular functions [10,11]. Recipient cells internalize exosomes *via* multiple endocytic pathways, including clathrin-mediated endocytosis, macropinocytosis, and lipid raft-mediated uptake [8]. After internalization, exosomes release their cargo into the cytoplasm or are routed to lysosomes for degradation. MicroRNAs (miRNAs) and proteins, profoundly influence recipient cells by modulating gene expression, regulating protein synthesis, and altering cellular signaling pathways [12]. These mechanisms allow exosomes to contribute to various biological processes, tissue repair, cancer progression, and immune regulation [13].

**Figure 1.**
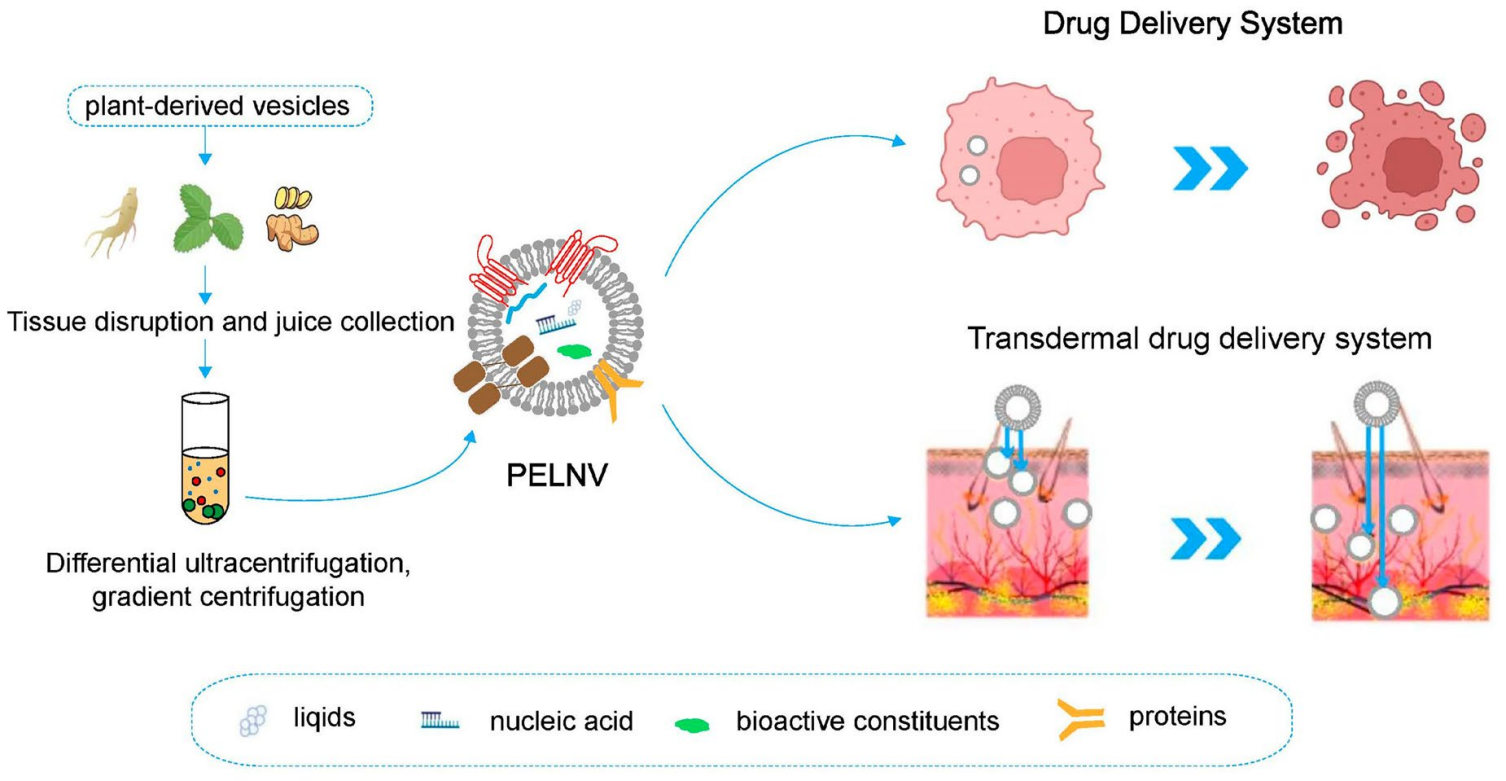
Schematic illustration of the isolation and application of plant-derived exosome-like nanovesicles (PELNVs) for drug delivery systems. Plant tissues undergo tissue disruption followed by differential ultracentrifugation and gradient centrifugation to isolate PELNVs. These vesicles encapsulate various bioactive cargo including lipids, nucleic acids, proteins, and other bioactive constituents. Their transdermal drug delivery schematic demonstrates ability to cross the epidermis and reach deeper dermal layers, highlighting their potential for enhanced skin therapies. Adapted from reference [70] under the terms and conditions of a Creative Commons License ((https://creativecommons.org/licenses/by/4.0/).

Plant-derived exosome-like nanovesicles (PDELNs) are nanoscale vesicles derived from plants that are structurally and functionally similar to mammalian exosomes. Despite containing lipid bilayer with proteins, nucleic acids, and other bioactive molecules, the exact biogenesis pathways of PDELNs are not well characterized. In plants, the formation of extracellular vesicles may use several pathways including MVB fusion with the plasma membrane and also may include other pathways like exocyst-positive organelle (EXPO) pathway and vacuole-associated secretion which are not equivalent to the canonical mammalian exosome biogenesis pathways. Mammalian exosomal surface protein markers are not broadly recognized as exosomal surface proteins in plant vesicles, nor MISEV guidelines (originally applied to characterize mammalian exosomes) [14]. In recent years, PDELNs have attracted considerable interest due to low immunogenicity and ability to transfer bioactive plant compounds to human cells [15,16]. This lower immune response not only improves their safety but also makes them better suited for long-term therapeutic applications. This feature renders PDELNs providing natural and biocompatible vehicle for drug delivery, especially in managing inflammatory skin diseases [17–21]. PDELNs are also natural compounds that are biocompatible, thus lowering the chances of adverse effects; hence, they are the ideal compounds to apply in therapeutic applications in the long-term. PDELNs have access to a wide range of bioactive molecules including microRNAs, proteins, phytochemicals, and thus can be used to target a variety of biological pathways, including inflammation modulation, skin repair, and regenerative processes [22]. Consequently, PDELNs have a great potential for natural and effective drug delivery vehicles, especially in the management of inflammatory skin diseases, such as psoriasis, atopic dermatitis, and chronic wounds (Figure 1) [23]. Moreover, they have been shown to be versatile in attacking different layers of the skin and different cell types, which increases their possible application in the area of skin regeneration and wound healing [24].

The outermost layer of the skin, the Stratum Corneum, is one of the most significant barriers of efficient skin delivery of nanosystems including vesicles or hydrogel-embedded therapeutic agents due to blocking large or hydrophilic molecules. Microneedles (MN) form micro channels in the skin circumvent the stratum corneum and allow deeper delivery of vesicles. Indicatively, the same review demonstrates that MN-mediated delivery of extracellular vesicles has considerably better regenerative performance than topical delivery due to greater penetration and retention in the dermis [25,26]. Electrospun nanofibre mats augment skin delivery by strongly resemble the extracellular matrix, can conform to irregularities in the skin surface, and allow active vesicles or hydrogels to be released to promote wound dressing and topical delivery because of high porosity and flexibility [27,28]. The third approach is iontophoresis (delivery of weak electric current) that can be used together with microneedle-assisted microchannels and cause the charged drugs molecules or nano-carriers to be pushed further into the skin. The use of MNs in conjunction with iontophoresis in a wearable patch format has recently been reported to enhance the transdermal delivery of model drugs to a considerable degree [29,30].

The purpose of the review is to find out the potential of the use of PDELNs and hydrogel systems in dermatology. It will also explore the role of the special molecular cargo of PDELNs that are flavonoids and microRNAs in improving skin regeneration over conventional therapies. The review also seeks to review the current developments in the combination of PDELNs with hydrogels with an emphasis on sustained release and release targeting. In addition, it will comment on new methods, including microneedles, electrospun fibers, and iontophoresis, to increase skin penetration. Finally, an overview of regulatory issues and suggestions to overcome these obstacles is going to be provided, as well as the future trends of the optimization of PDELN-based therapies in dermatology.

## 2. Isolation and purification methods of PDELNs

Recent technological advances improved isolation and purification of PDELNs. Common methods like differential ultracentrifugation, size-exclusion chromatography, and advanced microscopy help characterize their size, composition, and function. While each technique has its own strengths and limitations [31].

### 2.1. Ultracentrifugation

Differential ultracentrifugation is most widely used technique for isolating PDELNs. This method involves spinning sample through several centrifugation steps at increasing speeds to remove cellular debris and larger vesicles. Initially, slower spins clear out the bigger contaminants, followed by higher-speed spins. Despite its popularity due to being simple and cost-effective, this method often results in co-isolation of protein aggregates and other vesicular impurities, which can reduce purity of final preparation [32].

### 2.2. Density gradient centrifugation

Density gradient centrifugation offers a more refined approach than standard ultracentrifugation by improving purity of isolated PDELNs. This method involves layering a density gradient medium, usually sucrose or iodixanol, during high-speed centrifugation. Although sucrose gradients have been traditional choice, iodixanol is gaining preference because it better preserves exosome structure and biological function under iso-osmotic conditions. Studies suggest that sucrose may compromise certain exosomal bioactivities, while iodixanol helps maintain both their structural integrity and functional properties. During centrifugation, PDELNs separate based on their buoyant densities within gradient, allowing for more precise separation from contaminating vesicles. Despite offering higher purity, this technique is more labor-intensive and demands careful handling with challenges in distinguishing PDELNs from particles with similar densities, such as microvesicles or viral particles [33].

### 2.3. Polymer-based precipitation

Polymer-based precipitation provides a scalable method for isolating PDELNs. This technique involves adding hydrophilic polymers like polyethylene glycol (PEG) to the sample, which excludes water molecules and causes PDELNs to precipitate. While this approach is user-friendly and easily scalable but often leads to the co-precipitation of non-exosomal extracellular materials, potentially compromising purity of isolated vesicles [34].

### 2.4. Size-exclusion chromatography

Size-exclusion chromatography (SEC) is a mild and efficient method that separates particles based on size, preserve delicate structure of PDELNs. In this process, the sample moves through a porous column, where larger particles elute first, followed by smaller ones. This size-based sorting enables isolation of PDELNs with minimal structural damage, which is often a concern with high-speed centrifugation. SEC is also commonly combined with other purification techniques to boost overall isolation quality and support further structural and morphological analysis. However, its limitation is inability to completely remove protein contaminants that are similar in size to PDELNs, which can reduce purity of final sample [35].

Although PDELNs hold potential for drug delivery, turning that into large-scale, practical applications is not without challenges. One of the major hurdles is achieving consistently high yields of pure PDELNs from plant sources, especially when working with large volumes. To address this, researchers have started using plant cell culture systems for example, in *Catharanthus roseus*, where cell cultures produced exosome-like vesicles in higher quantities compared to direct extraction from tissues. This approach not only improves yield but also helps minimize variability caused by environmental or seasonal factors. Advanced techniques like tangential flow filtration emerging as scalable and gentle alternatives for exosome isolation, especially when combined with established methods. These approaches help preserve exosomal structure and function. Researchers have also observed that adjustments in early extraction steps such as selecting appropriate buffers or using gentler handling protocols can influence both yield and quality of final product. In parallel, innovations like microfluidic platforms and field-flow fractionation are gaining attention for their ability to achieve more precise separation and reduce the presence of non-exosomal contaminants that often remain in conventionally processed samples. To ensure reliability and safety particularly in therapeutic contexts, there is growing emphasis on standardized quality control measures, including adherence to international frameworks like the Minimal Information for Studies of Extracellular Vesicles (MISEV) guidelines [36].

Microfluidic systems have become both highly controllable and compact in nature: e.g. a double-filtration microfluidic device has allowed rapid isolation of PDELNs (50–100 uL plasma real-time in less than 50 min) of similar size and miRNA content to those with polyethylene-glycol, yet with much greater purity and capability to operate in point of care [37–39]. In the meantime, bioreactor systems, in particular hollow fibre 3D culture cartridges, have demonstrated enhanced yield by orders of magnitude and scalable functionality. As an example, cultivated mesenchymal stromal cells (MSCs) in a hollow-fibre bioreactor generated up to approximately 38-fold greater extracellular vesicle harvests than conventional flask-culture with preservation of size, phenotype, RNA-cargo and in-vivo efficacy [40,41]. These developments can be considered to have dealt with two key bottlenecks: (a) the requirement that high-throughput production is maintained in a form that can be used to manufacture clinical-grade therapy; and (b) the decrease in process variability and cost per dose (through closed, continuous culture, reduced use of lower medium and integrated harvesting) [42,43]. Combined with the developments in microfluidic isolation and bioreactor-based production platforms, integration is becoming a feasible step towards GMP-compatible production of PDELNs/hydrogel systems on large-scale levels with respect to dermatological and regenerative medicine applications.

## 3. Comparison of PDELNs with alternative nanocarriers

PDELNs are increasingly being explored as an alternative to mammalian-derived exosomes (MDEs) in dermatological therapies, owing to their unique biogenesis, distinct molecular cargo, and practical advantages for clinical use. Unlike MDEs, which are isolated from cell cultures or biological fluids through complex and costly procedures, PDELNs can be obtained more easily from common plant sources, making the process more scalable and cost-effective [44,45]. Structurally, PDELNs carry unique phytochemicals, including flavonoids, microRNAs, and antioxidants, which confer intrinsic anti-inflammatory and regenerative properties beneficial for skin repair [46]. Synthetic nanocarriers often require cryoprotectants or chemical stabilizers to maintain integrity during storage and biological transit, which adds complexity and cost to formulation process [47]. Moreover, PDELNs exhibit low immunogenicity and reduced risk of zoonotic pathogen transmission compared to MDEs, thereby improving their safety profile [48]. These features make PDELNs particularly suitable for integration into advanced drug delivery platforms such as hydrogels, enabling enhanced stability, targeted delivery, and sustained release of therapeutic payloads [45,49]. Intrinsic biological makeup of PDELNs contributes to their exceptional biocompatibility and minimal immunogenicity. For instance, PEGylated nanoparticles, although commonly used, have been shown to induce anti-PEG antibody production, leading to rapid clearance and reduced therapeutic efficacy [50]. Nanoparticles often require surface engineering, such as ligand conjugation, to achieve similar targeting capabilities, which can unpredictably affect pharmacokinetics and biodistribution [51]. In contrast to synthetic drug carriers, which are typically limited to delivering a single therapeutic agent, PDELNs carry diverse range of bioactive molecules, including miRNAs, lncRNAs, proteins, lipids, and secondary plant metabolites that can work synergistically. This multifaceted cargo enables them to influence multiple biological pathways at once, including those involved in inflammation, oxidative stress, and tissue repair [52]. The integration of artificial intelligence (AI) and machine learning into the development of PDELN-based formulations offers fine-tuning of critical parameters such as vesicle size, surface charge, cargo loading efficiency, and release kinetics, thereby accelerating precision of personalized dermatological therapies tailored to specific skin conditions [53]. Use of PDELNs in the cosmetic and skincare industries is rapidly expanding, driven by their natural origin, low toxicity, and ability to deliver active plant-derived compounds directly into the skin. These features align well with the growing demand for safe, effective, and sustainable cosmeceutical products [54].

The PDELNs present a unique molecular cargo, including flavonoids, microRNAs (miRNAs), proteins and lipids, which makes them unique in comparison to traditional carriers and provides them with higher skin regeneration. As an example, a comparison between keratinocyte transcriptome response identified that ginseng- and green-tea-derived PDELNs caused much more changes in gene-expression pathways associated with barrier formation, collagen synthesis and skin regeneration when compared to identical green-tea plant extracts [52,55]. Intrinsic antioxidant and anti-inflammatory actions of the flavonoid-rich tea leaf vesicles containing epigallocatechin gallate (EGCG) and quercetin also decrease oxidative stress in dermal cells and contribute to tissue repair. Besides, PDELNs contain miRNA with the capacity to regulate gene-expression pertinent to extracellular matrix remodelling and cell migration; in case of lavender, miRNA 166 3p PDELNs were demonstrated to govern collagen metabolism in the photo-aged skin models [52]. Overall, the synergistic bioactivity of bioactive small molecules and nucleic acid cargo in PDELNs stimulates a multifunctional regenerative response, including antioxidation + immunomodulation + gene regulation, more potent than numerous conventional single-agent systems, which depend on a single mechanism of action.

## 4. Mechanistic insights of PDELNVs in dermatology

The human skin represents barrier to therapeutic delivery, primarily due to the stratum corneum, which restricts the permeation of hydrophilic and high-molecular-weight compounds. To address this limitation, nanotechnology-based delivery systems such as PDELNs have emerged as novel platforms [23,56]. These vesicles, owing to their nano-size, lipid bilayer structure, and natural origin, are capable of navigating the intercellular lipid matrix of the skin. However, the delivery efficiency of PELNVs alone remains limited by rapid degradation and poor retention at the application site [57]. Hydrogels improve the stability of PDELNs, enable sustained release, and promote deeper diffusion across the skin layers by maintaining hydration and modulating the local microenvironment. This dual system has shown efficacy in wound healing, anti-inflammatory therapy, and skin regeneration. Moreover, future strategies integrating microneedle arrays and phototherapy have been proposed to further disrupt the skin barrier in a controlled manner and increase the transdermal penetration of bioactive cargos [58]. Nonetheless, the combined PDELNs–hydrogel platform represents a state-of-the-art nanotechnology-based enhancer with potential to overcome intrinsic skin penetration barriers [59].

**Figure 2.**
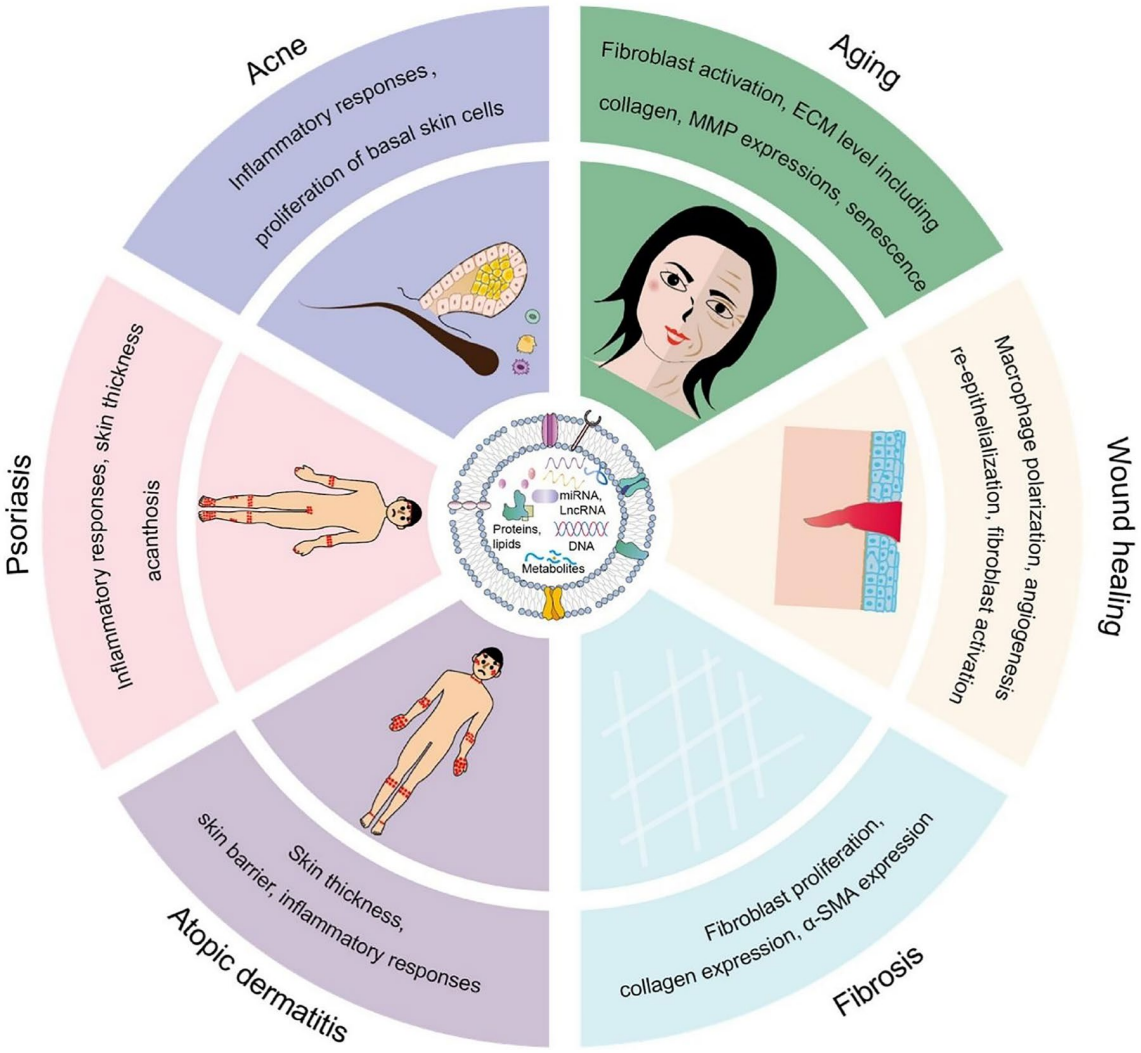
Schematic representation of PDELN architecture and its cargo of bioactive molecules. These are surrounded by major skin disorders and pathological processes which are regulated by PDELNs. PDELNs affect inflammatory reactions and basal cells growth in acne. They influence fibroblast activation, extracellular matrix remodelling, matrix metalloproteinase and cellular senescence in aging skin. PDELNs stimulate the polarization of macrophages, angiogenesis, re-epithelialization, and activation of fibroblasts during wound healing. They also control the fibroblast growth, collagen synthesis, and a-smooth muscle actin in fibrosis. In atopic dermatitis, PDELNs would be involved in the control of skin barrier function, thickness, and inflammatory responses. In psoriasis, they are directed to inflammatory pathways, skin thickening, and acanthosis. Adapted from reference [67] under the terms and conditions of a creative commons license (https://creativecommons.org/licenses/by/4.0/).

PDELNs interact with skin cells through multiple mechanisms (Figure 2), having capacity to deliver functional RNA molecules like microRNAs and mRNAs to target cells like keratinocytes and fibroblasts [60]. PDELNs carrying specific microRNAs can modulate the expression of genes. MRNAs deliver and support protein synthesis, contributing to restoration of normal skin function [61]. PDELNs have been shown to contain various bioactive proteins, such as growth factors and cytokines, which play important roles in skin cell repair and regeneration [62]. For instance, it carry epidermal growth factor (EGF) or fibroblast growth factors (FGFs) can promote keratinocyte proliferation and fibroblast migration for wound healing and restoring skin’s barrier function [63].

PDELNs regulate inflammatory pathways of skin, which are frequently disrupted in disease disorders [64]. Skin inflammation is controlled by nuclear factor kappa B (NF-κB), interleukin-6 (IL-6), and tumour necrosis factor-alpha (TNF-α) [65]. These cytokines involve in promoting inflammation, tissue damage, and disrupting the skin’s barrier function. Research has shown that PDELNs can modulate these inflammatory pathways, potentially reduce skin inflammation and slow the progression of chronic skin conditions. PDELNs inhibit the activation of NF-κB, a transcription factor that controls the expression of several pro-inflammatory genes, including IL-6 and TNF-α. By targeting these pathways, PDELNs may help to alleviate skin’s inflammatory response [66]. PDELNs interact with epidermal and dermal layers of the skin, affecting important physiological processes involved in skin barrier function and wound healing. Dermal barrier consists of keratinocytes in epidermis and extracellular matrix components in as body’s first defense against environmental damage, pathogens, and moisture loss [67]. Damage to barrier leads to different skin conditions in atopic dermatitis and chronic wounds. Studies suggest that PDELNs may strengthen skin barrier integrity by encouraging the production of tight junction proteins and promoting keratinocyte differentiation [68]. Furthermore, PDELNs have demonstrated the ability to accelerate wound healing by enhancing the migration and proliferation of key skin cells, especially fibroblasts and keratinocytes, which are vital for tissue regeneration. By interacting with these skin layers, PDELNs may contribute to restoring normal skin structure and promoting recovery in damaged or diseased tissue [23,56,69]. These vesicles, owing to their nano-size, lipid bilayer structure, and natural origin, are capable of navigating the intercellular lipid matrix of the skin. However, the delivery efficiency of PELNVs alone remains limited by rapid degradation and poor retention at the application site [57]. Hydrogels improve the stability of PDELNs, enable sustained release, and promote deeper diffusion across the skin layers by maintaining hydration and modulating the local microenvironment [58,59].

## 5. Role of PDELNs for dermatological disorders

Unique properties of PDELNs make them ideal candidates for addressing key challenges in dermatology, including inflammation modulation, immune regulation, and tissue repair [70].

### 5.1. Immunomodulatory and anti-inflammatory effects of PDELNs

PDELNs have shown remarkable potential in modulating immune responses and controlling inflammatory pathways in skin disorders such as psoriasis is a chronic, immune-mediated dermatosis marked by epidermal hyperplasia and systemic inflammation, primarily driven by the dysregulation of Th17 cell signalling. Elevated levels of IL-17, IL-22, and TNF-α contribute to keratinocyte proliferation, resulting in the formation of characteristic psoriatic plaques [71]. Recent evidence indicates that PDELNs can modulate these key inflammatory pathways by delivering bioactive cargo such as anti-inflammatory microRNAs and immunoregulatory proteins. Specifically, PDELNs have been shown to downregulate Th17-associated cytokines, thereby attenuating the inflammatory loop central to psoriasis pathogenesis [72]. In addition to immunomodulation, PDELNs exert a direct effect on keratinocytes, promoting normalization of cell-cycle dynamics and differentiation processes, which helps mitigate epidermal hyperproliferation and scaling [73]. Majority of findings are derived from *in vitro* experiments and animal models of psoriasis, and robust randomized clinical trials evaluating PDELNs in human psoriatic patients remain limited [74].

Moreover, atopic dermatitis (AD) is a chronic, relapsing inflammatory skin condition that mainly affects children but often persists into adulthood. The pathogenesis of AD involves a multifaceted interaction between genetic predisposition, skin barrier impairment, immune dysregulation, and environmental influences. Hallmark of AD is the elevated expression of Th2-associated cytokines, notably interleukin (IL)-4, IL-5, and IL-13, which contribute to eosinophilic inflammation and impaired skin homeostasis [16,75]. Recent studies highlight the regulatory role of non-coding RNAs (ncRNAs), such as microRNAs and long non-coding RNAs, in modulating barrier protein expression, cytokine signalling, and inflammatory cascades in AD. Although bioinformatics analyses have identified multiple dysregulated ncRNAs in AD lesions, their precise functional mechanisms remain incompletely understood. PDELNs offer a novel, biologically active nanocarrier system capable of delivering regulatory microRNAs and proteins directly to target skin cells. PDELNs have been shown to suppress Th2 cytokine expression, particularly IL-4 and IL-13, thereby reducing local inflammation and restoring immune balance. Moreover, PDELNs enhance the expression of structural proteins such as filaggrin and involucrin, which are essential for reconstructing epidermal barrier function and preventing trans epidermal water loss. In addition, anti-pruritic effects have been observed, attributed to the downregulation of itch-associated cytokines, offering multifaceted symptomatic relief. These mechanistic actions position PDELNs as adjuncts or alternatives to conventional AD therapies, especially in pediatric and steroid-sensitive populations [76,77]. Rosacea is chronic, relapsing inflammatory dermatosis predominantly affecting the central face, characterized clinically by persistent erythema, telangiectasia, papules, and pustules. Pathophysiology involves complex interplay of immune dysregulation, neurovascular abnormalities, and microbial factors. Increased expression of IL-1β, TNF-α, and vascular endothelial growth factor (VEGF) plays an important role in mediating the persistent inflammation and abnormal vascular responses [78]. PDELNs have demonstrated potential in modulating underlying pathogenic mechanisms by selectively targeting immune cells involved in disease progression and reducing cytokine-mediated skin inflammation which limit formation of papules and pustules. This ability to regulate both inflammatory and vascular responses positions PDELNs for managing rosacea symptoms and preventing disease flare-ups. Their dual immunomodulatory and vascular-regulatory actions highlight the therapeutic versatility of PDELNs [79]. Evidence of these effects is mostly based on preclinical trials and the mechanism research, and properly designed clinical trials in AD patients are still limited [80].

Systemic lupus erythematosus (SLE) and cutaneous lupus erythematosus (CLE) represent complex autoimmune disorders frequently presenting with cutaneous manifestations, notably the characteristic malar or “butterfly” rash affecting the face. Pathogenesis involves autoantibody-mediated targeting of skin cells and extracellular matrix components, precipitating widespread inflammation and tissue injury. Central to disease progression is the dysregulated activation of T and B lymphocytes, accompanied by elevated production of type I interferons and pro-inflammatory cytokines [81]. PDELNs may confer therapeutic benefits by modulating aberrant immune responses and attenuating autoimmune-driven inflammation. These vesicles can deliver specific microRNAs that influence immune cell differentiation and effector functions, thereby potentially mitigating the hyperactivation of T and B cells characteristic of lupus. The theoretical advantages of PDELNs in lupus are indirectly immunomodulatory in nature and specific clinical trials in SLE or CLE cohorts have not yet been developed [82].

### 5.2. Skin regeneration and wound healing potential of PDELNs

The regenerative capabilities of PDELNs play a vital role in wound healing and tissue repair processes, particularly in chronic wounds and post-surgical recovery. Chronic wounds, including diabetic ulcers and pressure sores, remain a major therapeutic challenge in dermatology due to impaired tissue regeneration and delayed healing. These conditions are typically characterized by deficient fibroblast migration, reduced angiogenesis, and suboptimal extracellular matrix (ECM) remodelling, all of which contribute to prolonged wound persistence. PDELNs have emerged as biotherapeutics capable of modulating multiple phases of the wound healing cascade. Specifically, PDELNs have been shown to enhance fibroblast proliferation and migration, which are essential for ECM deposition and granulation tissue formation. Furthermore, these nanovesicles stimulate angiogenesis critical process for restoring vascularization—by delivering bioactive cargo such as vascular endothelial growth factor (VEGF) and transforming growth factor-beta (TGF-β) [83,84]. Although the molecular structure of plant-derived VEGF and TGF-β analogues may differ from their mammalian counterparts, experimental data suggest they exert comparable pro-regenerative effects in target cells. This includes the activation of pro-healing signalling pathways, enhancement of collagen synthesis, and acceleration of epithelial closure. PDELNs facilitate coordinated tissue repair by targeting cellular, molecular, and vascular components of the wound bed, thereby improving both the rate and quality of healing. These findings underscore the potential of PDELNs as next-generation wound therapeutics, particularly in chronic and refractory wound types [62,85]. For example, MEMC-Gel dressing combining plant-derived and stem cell exosomes exhibited a controlled degradation profile alongside gradual exosome release, leading to accelerated wound closure in diabetic mice [59]. Recent clinical observations from a retrospective case-series on rose stem cell-derived exosomes (RSCEs) provide compelling quantitative and qualitative evidence for their role in scar remediation and wound healing. In this series, four cases with varying scar aetiologies including trauma, burn, laser injury, and dog-bite wounds were treated over 2–4 months with RSCEs applied topically, with microneedling, laser therapy, or alone. Across these cases, objective improvements were assessed *via* standardized scar scales: the modified Vancouver Scar Scale (mVSS) scores improved by approximately 70-80%, while both observer and patient scores on the Patient and Observer Scar Assessment Scale (POSAS) increased by roughly 60-70%. Notably, in one laser-injured scar managed with only topical RSCEs, mVSS declined and POSAS also improved robustly, emphasizing that even non-device-based applications can achieve substantial clinical benefit. Improvements were consistent in scar elevation, texture, coloration, and overall integration with surrounding skin, with no adverse effects reported [86]. The synergistic effect of *Plantago major* extract combined with laser therapy enhances fibroblast migration and modulates key cytokines (IL-6, TNF-α) and growth factors (VEGF), promoting efficient wound repair. This combinatorial approach exemplifies how traditional medicinal compounds and modern photodynamic techniques can jointly influence cellular mechanisms involved in skin regeneration [87].

Acne vulgaris is a multifactorial inflammatory skin disorder characterized by the obstruction of sebaceous follicles, leading to the formation of comedones, papules, pustules, and, in severe cases, nodulocystic lesions. The pathogenesis involves excessive sebum production, altered follicular keratinization, and an inflammatory response driven by Cutibacterium acnes colonization. PDELNs have emerged as a novel therapeutic strategy for acne management due to their ability to modulate both sebaceous activity and cutaneous inflammation. MicroRNAs and proteins encapsulated within PDELNs can regulate the expression of genes associated with sebocyte differentiation and lipid synthesis, thereby potentially reducing sebum overproduction and limiting follicular blockage [88]. PDELNs exert anti-inflammatory effects by downregulating key cytokines implicated in acne pathophysiology, including tumor necrosis factor-alpha (TNF-α), interleukin-1β (IL-1β), and IL-8. This cytokine suppression may mitigate neutrophilic infiltration and lesion progression, accelerating the resolution of active acne. By simultaneously targeting lipid dysregulation and immune-mediated inflammation, it offers a multi-pronged approach to acne therapy and holds potential as biocompatible alternatives to conventional pharmacological agents, especially in cases with antibiotic resistance or sensitivity to retinoids [89]. PDELNs evidenced managing acne in preclinical studies. There are limited data of clinical trials involving their use in the treatment of acne in a human population [90]

Skin cancers, including melanoma and non-melanoma forms basal cell carcinoma and squamous cell carcinoma, which developed due to uncontrolled cellular proliferation driven by mutations, often resulting from environmental exposures like ultraviolet (UV) radiation. The tumor microenvironment in these cancers is characterized by chronic inflammation, with various immune cells and cytokines contributing to tumour growth, invasion, and metastasis [91]. PDELNs present therapeutic potential in the context of skin cancer through their ability to modulate immune responses and inhibit tumour progression. PDELNs may impede the metastatic potential of cancer cells by regulating key processes including cell adhesion, migration, and extracellular matrix remodelling. By targeting these essential pathways, PDELNs have the capacity to retard tumor progression, enhance anti-tumour immunity, and facilitate the restoration of skin integrity post-tumor resection [92]. Eczema herpeticum is rare but associated with complication of atopic dermatitis caused by herpes simplex virus type 1 (HSV-1) infection. It presents with groups of painful vesiculopustular lesions, often accompanied by fever, malaise, and extensive skin involvement. This condition may lead to secondary bacterial infections, scarring, and potentially systemic spread of virus [93]. Given dual inflammatory and viral nature of eczema herpeticum, PDELNs have emerged as adjunctive therapy. PDELNs may bolster the host’s antiviral defenses by delivering immune-regulatory microRNAs and cytokines that activate antiviral pathways, including interferon signalling [94]. The anti-tumour effects above are mostly backed by experimental and preclinical research and clinical translation of these effects in melanoma or non-melanoma skin cancers is in its early stages of research [95].

### 5.3. Addressing skin ageing and hyperpigmentation with PDELNs

PDELNs offer a dual approach to improving the appearance and health of aging skin by stimulating structural protein production and mitigating oxidative stress. Aging skin is characterized by a progressive reduction in the synthesis of collagen, elastin, and other key extracellular matrix components, resulting in wrinkles, diminished elasticity, and dermal thinning. This degenerative process is further exacerbated by heightened oxidative stress, chronic low-grade inflammation, and the accumulation of senescent cells, which collectively accelerate the manifestation of visible aging signs. These multifactorial changes impair skin structural integrity and function, thereby contributing to the characteristic phenotypes of aged skin [96]. PDELNs have been recognized for their potential in skin rejuvenation, primarily through the targeted delivery of growth factors, proteins, and microRNAs that regulate collagen synthesis and elastin production. PDELNs, in particular, have been shown to stimulate dermal fibroblasts, thereby enhancing collagen deposition, improving skin elasticity, and reducing the visible signs of aging like wrinkles [54,97]. In addition to their regenerative properties can mitigate oxidative stress by transporting antioxidant molecules that neutralize free radicals, thereby protecting skin cells from oxidative damage. Through the dual modulation of structural protein synthesis and redox balance, PDELNs hold potential in reducing visible signs of skin aging and promoting overall skin health and rejuvenation [54]. Majority of anti-aging effects of PDELNs are still presented in small scale exploratory studies and there have not established standardized clinical trials that determine long-term cosmetic and dermatological outcomes of these compounds [98].

Hyperpigmentation disorders, including melasma and post-inflammatory hyperpigmentation (PIH), are characterized by localized or diffuse skin darkening caused by excessive melanin synthesis and deposition. Melasma is often linked to hormonal fluctuations during pregnancy or oral contraceptive use, whereas PIH develops inflammatory skin insults or trauma, including acne, eczema, or burns [99]. PDELNs demonstrate potential therapeutic effects in managing hyperpigmentation disorders by modulating melanogenic signalling pathways. By delivering functional microRNAs and regulatory proteins, PDELNs can inhibit key melanogenic enzymes, tyrosinase, thereby suppressing melanin synthesis. These vesicles may alleviate oxidative stress and inflammatory responses, both of which contribute to the exacerbation of hyperpigmentation. Through simultaneous regulation of melanocyte function and inflammatory pathways implicated in post-inflammatory hyperpigmentation, PDELNs offer novel strategy to skin lightening and tone improvement [54].

### 5.4. Targeting specific dermatological disorders using PDELNs

PDELNs are versatile in targeting specific skin conditions, offering potential for novel treatments where conventional therapies may fall short. Vitiligo is a chronic depigmenting skin disorder marked by the progressive loss of functional melanocytes, resulting in well-demarcated hypopigmented patches on the skin. The etiopathogenesis is multifactorial, with strong evidence pointing toward autoimmune-mediated melanocyte destruction, compounded by oxidative stress, genetic susceptibility, and pro-inflammatory cytokine signalling [100]. PDELNs may exert immunomodulatory effects by downregulating autoimmune responses against melanocytes. This is achieved through the delivery of anti-inflammatory cytokines and regulatory microRNAs that can inhibit T-cell-mediated cytotoxicity and alter the expression of MHC class I/II molecules on melanocytes. Importantly, some plant-derived vesicles also contain antioxidant phytochemicals capable of neutralizing reactive oxygen species (ROS), thereby protecting melanocytes from oxidative damage another key factor in vitiligo progression. By simultaneously addressing immune dysregulation, oxidative stress, and melanocyte regeneration, PDELNs present a multi-targeted therapeutic strategy with high translational potential for non-invasive vitiligo [101]. Novel therapeutic options in vitiligo are still at preclinical stage, and specific human clinical trials are yet to be established [95].

Seborrheic dermatitis is a chronic inflammatory dermatosis that commonly affects the scalp, face, and upper trunk, and is clinically marked by erythema, flaking, and greasy, yellowish plaques. The condition is closely associated with colonization by *Malassezia* species—a lipophilic yeast known to aggravate inflammation, particularly in genetically predisposed individuals. Its pathogenesis involves a dysregulated immune response, characterized by elevated levels of pro-inflammatory cytokines, including interleukin-1β (IL-1β), IL-6, and tumor necrosis factor-alpha (TNF-α), along with hyperactivity of the sebaceous glands [102]. PDELNs exert a dual mechanism of action by simultaneously targeting the inflammatory microenvironment and the dysregulation of sebaceous gland activity that contribute to the pathogenesis of seborrheic dermatitis [67]. PDELNs have not been specifically analysed in clinical evidence and additional human research to support the suggested mechanisms is needed [103]. Psoriatic arthritis (PsA) is chronic autoimmune disorder characterized by co-occurrence of psoriatic skin lesions and inflammatory arthritis involving peripheral joints. The pathogenesis of PsA involved immune dysregulation, Th17 cells and pro-inflammatory cytokines, IL-17, TNF-α, and IL-23, which collectively contribute to initiation and progression [104]. By modulating Th17 cytokine axis and associated downstream inflammatory pathways, PDELNs have the potential to alleviate both the cutaneous and articular symptoms of psoriatic arthritis. PDELNs may regulate activity of matrix metalloproteinases (MMPs), enzymes involved in degradation of joint tissues, thereby supporting tissue repair and potentially preventing structural joint damage [105]. The potential benefits in psoriatic arthritis are primarily inferred from immunomodulatory effects observed in preclinical systems, and clinical validation in PsA patients is currently insufficient [74].

## 6. Hydrogels as drug delivery systems

Hydrogels are three-dimensional crosslinked polymer matrices capable of extensive water retention, conferring mechanical compliance and hydration that closely mimic native soft tissues, thereby enhancing their suitability for dermatological and soft tissue applications [106]. Natural hydrogels synthesized from polysaccharides (alginate, hyaluronic acid, chitosan) or proteins (gelatin, collagen) continue to dominate preclinical and clinical research because they offer excellent biocompatibility, inherent biodegradability, and often possess bioactive cues inherent to the extracellular matrix. For instance, chitosan hydrogels have been modified to enhance skin adhesion, antimicrobial activity, and to adjust release rates through variable crosslinking density or composite inclusion (e.g. with nanoparticles) in recent reports [107]. Synthetic systems such as PNIPAAm-based thermoresponsive hydrogels, PEG-based hydrogels, and PLGA-PEG-PLGA triblock copolymers have been engineered for precise control of sol-gel transition temperature, drug loading, and sustained release, though often at the trade-off of decreased biodegradability and potential cytotoxicity unless appropriately modified [108]. Smart hydrogels responsive to pH, temperature, or even enzyme presence are increasingly studied for transdermal or wound healing applications to allow on-demand drug release [109].

### 6.1. Hydrogel mechanisms in drug delivery

**Figure 3.**
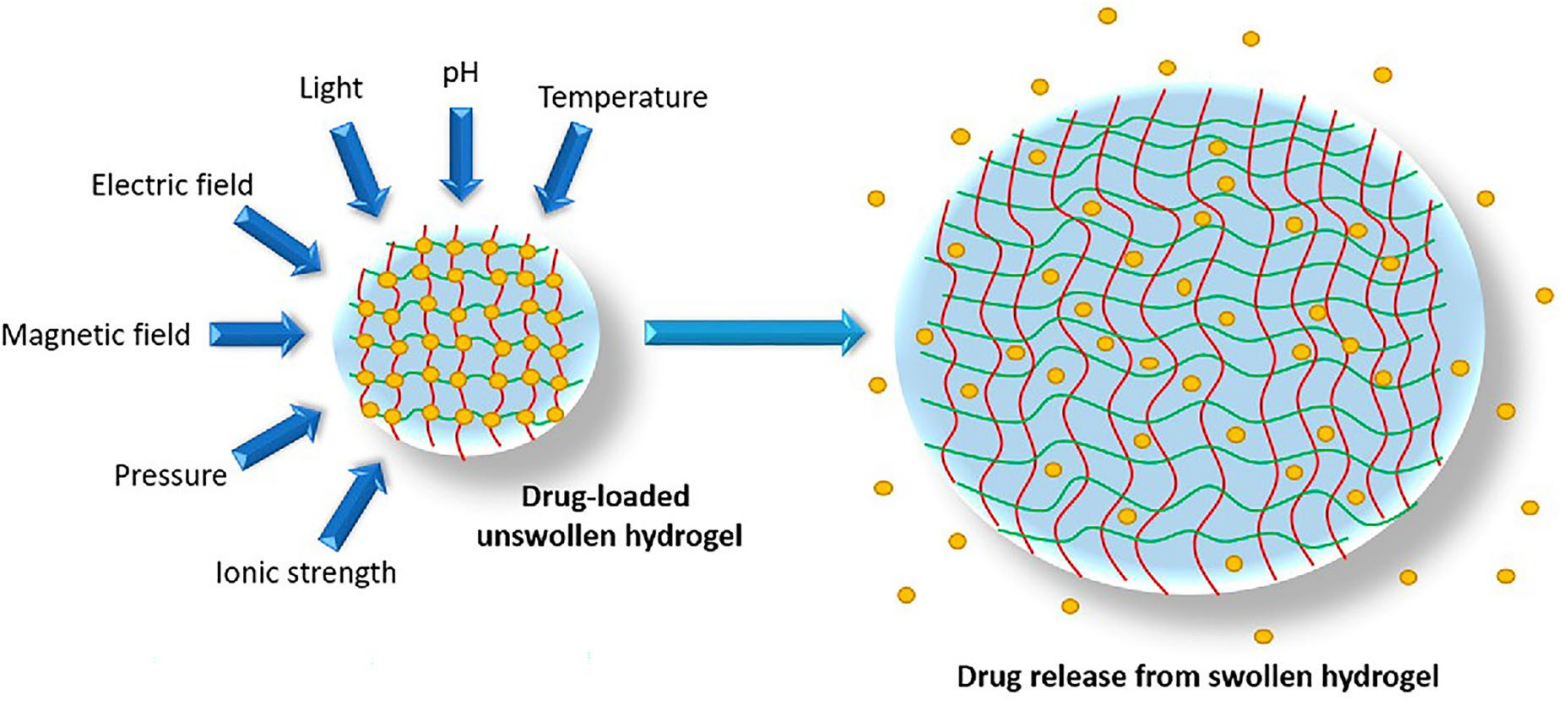
Swelling of a drug delivery hydrogel in response to chemical and physical stimuli, with red and yellow lines representing the matrix structure and yellow dots denoting drug molecules. Adapted from reference [159] under the terms and conditions of a creative commons license (https://creativecommons.org/licenses/by/4.0/).

Hydrogels facilitate the controlled and sustained release of therapeutic agents (Figure 3) by exploiting various mechanisms, including diffusion-controlled release, pH-sensitive release, and thermo-responsive behavior [110]. These systems are primarily driven by the interaction between the hydrogel matrix and the surrounding environment, with drug release influenced by several critical factors, including the polymer network’s crosslink density, swelling properties, and external stimuli [111]. Diffusion-controlled release constitutes a primary mechanism in hydrogel-based drug delivery systems, wherein drug molecules gradually diffuse from the hydrogel matrix into the external environment. The release kinetics are influenced by parameters including the molecular size of the drug, the swelling characteristics of the hydrogel, and the microarchitecture of the polymer network [112,113]. Swelling of hydrogels forms channels that enable controlled diffusion, supporting prolonged therapeutic release at the target site [112].

### 6.2. Stimuli-responsive behaviour of hydrogels

The pH value of the skin and wound microenvironment is highly dynamic and varies throughout the stages of wound healing, impacting factors such as microbial activity, enzyme function, oxygen diffusion, and cellular behaviour. While intact skin maintains a slightly acidic surface pH (4–6), injury exposes internal tissues with a more neutral to alkaline pH (7.4). During normal healing, wounds typically transition from alkaline to acidic; however, in chronic or diabetic wounds, prolonged inflammation often maintains an alkaline environment (pH >7.3), promoting bacterial colonization and delaying healing. As such, designing smart delivery systems that respond to these fluctuating pH levels has become increasingly important in dermatological therapeutics [114]. Incorporating PDELNs into pH-responsive hydrogel systems offers site-specific and stage-specific delivery of bioactive molecules. Given the natural bioactivity of PDELNs and their ability to modulate inflammatory cytokines and support tissue regeneration, integrating them within hydrogels engineered to respond to pH gradients found in chronic wounds could accelerate healing, especially in diabetic skin ulcers [115,116].

Temperature plays a critical role in skin physiology and wound healing, as it directly influences enzyme activity, blood flow, and inflammatory responses. Clinical studies have shown that elevated periwound temperature is associated with better healing outcomes, while sudden increases can also indicate infection or inflammation. These variations present an opportunity for designing temperature-responsive hydrogel systems that dynamically respond to skin temperature changes [117]. Such hydrogels exhibit a sol–gel phase transition near physiological temperatures, allowing them to conform to irregular wound geometries and enable sustained, localized drug release. In the context of PDELNs delivery, thermosensitive hydrogels offer a unique advantage by enabling temperature-triggered release of cargos precisely when inflammatory or regenerative signals are active [117]. Thermo-responsive hydrogels are smart biomaterials that undergo a reversible sol–gel transition in response to temperature fluctuations. Typically, these hydrogels exhibit a lower critical solution temperature (LCST), above which they transition from a liquid (sol) to a gel state, enabling controlled encapsulation and release of bioactive agents. This temperature-dependent phase change alters the network structure, swelling behaviour, and diffusion kinetics of the hydrogel, thereby modulating the drug release profile. Such responsiveness can be strategically harnessed in dermatological applications, where local or systemic temperature changes—due to inflammation, infection, or external stimuli like photothermal therapy serve as natural triggers for release. When PDELNs are incorporated into thermo-sensitive hydrogel matrices, this sol–gel behaviour facilitates their sustained and spatially controlled delivery to skin tissues, particularly in conditions requiring dynamic dosing such as chronic wounds or inflammatory dermatoses. Moreover, by fine-tuning polymer composition, crosslinking density, and gelation thresholds, it is possible to customize the hydrogel’s thermal sensitivity to match physiological skin temperatures, thereby ensuring stability and therapeutic efficacy [118].

In dermatology, the occlusive nature of topical formulations is essential for preserving skin hydration by minimizing transepidermal water loss (TEWL) through barrier formation. This barrier mimics the function of the skin’s lipid matrix, preserving moisture within the stratum corneum and creating a hydrated microenvironment conducive to healing. Occlusion is especially effective in treating chronic inflammatory skin diseases like atopic dermatitis and psoriasis, where impaired epidermal barriers cause increased water loss and symptom exacerbation. Hydrogels, due to their semi-occlusive yet breathable nature, strike an optimal balance between hydration retention and skin permeability, making them ideal vehicles for the delivery of bioactive agents like PDELNs. Unlike petrolatum-based products that merely trap moisture, hydrogels can actively hydrate the skin while preserving its respiratory capacity—facilitating deeper penetration without compromising epidermal integrity. Recent clinical findings suggest that hydrogel-based formulations not only improve patient compliance due to their cooling and non-greasy properties but also enhance drug absorption by increasing SC hydration and modulating the hydrophilic–hydrophobic gradient across the skin barrier. For instance, studies comparing hydrogel vehicles to conventional lotions have demonstrated superior reductions in TEWL and improved skin moisture retention, effects that directly influence the diffusion and bioavailability of topically applied therapeutic agents. When coupled with the regenerative and anti-inflammatory properties of PDELNs, such hydrogel platforms offer a dual benefit restructuring the damaged barrier and delivering functional biomolecules efficiently to targeted skin layers [119,120].

The occlusive properties of hydrogels play a pivotal role in enhancing the therapeutic efficacy of PDELNs-based topical treatments, particularly in chronic inflammatory skin conditions such as psoriasis and atopic dermatitis. Occlusivity refers to the formation of a semi-permeable barrier over the skin surface, which limits transepidermal water loss (TEWL) and promotes sustained hydration within the stratum corneum. Unlike traditional occlusive agents like petrolatum that only create a physical seal, hydrogels offer a dual function—moisture retention and active delivery of therapeutic agents. When PDELNs are incorporated into occlusive hydrogel matrices, the enhanced hydration of the skin microenvironment can facilitate better permeation of these nanoscale vesicles through the stratum corneum. Hydrated skin exhibits lower resistance to diffusion, thus allowing exosomal contents such as anti-inflammatory miRNAs and proteins to reach deeper skin layers. Moreover, studies have demonstrated that hydrogel formulations reduce TEWL and increase epidermal hydration compared to conventional creams or lotions. This not only improves skin barrier function but also supports patient adherence due to the non-greasy, breathable, and soothing nature of hydrogels. The occlusive yet permeable structure of these carriers makes them an ideal platform for delivering bioactive PDELNs in dermatological therapies, especially where prolonged moisture and drug contact are essential for clinical efficacy [120,121].

### 6.3. Advancements in nanoemulsions for hydrogel-based skin delivery

Hydrogel-based drug delivery systems offer potential in treating skin conditions, though they face several inherent challenges that limit their efficacy. One primary issue is their restricted drug loading capacity. Due to the high-water content and crosslinked polymer network, hydrogels struggle to incorporate and retain therapeutic doses, especially those with hydrophobic properties or large molecular sizes. This constraint can negatively impact the amount of drug available for therapeutic delivery, which in turn may diminish effectiveness [116]. Another challenge lies in the control of hydrogel degradation rates. Ideally, hydrogels should degrade at a rate that matches the desired drug release profile; however, achieving such balance is difficult. Fast degradation can lead to rapid drug release, potentially causing inefficiencies in prolonged treatments, while slow degradation may delay drug delivery, limiting the therapeutic benefits over extended periods. A controlled degradation rate is crucial for providing sustained therapeutic effects, particularly in chronic skin diseases where continuous treatment is required [122]. Integrating nanoparticles into hydrogel formulations has emerged as novel strategy. Nanoparticles including liposomes, solid lipid nanoparticles, and metal nanocarriers offer superior drug encapsulation and enhance penetration through the skin barriers. Substituted surface properties facilitate targeted delivery to specific skin layers. Additionally, these systems enable controlled, sustained release of therapeutics, improving efficacy and reducing dosing frequency [123]. Nanoemulsions, colloidal dispersions of oil and water stabilized by surfactants, offer valuable approach. Their small droplet size improves drug solubility and stability, facilitating more efficient permeation through the skin barrier. When incorporated into hydrogel matrices, nanoemulsions synergistically enhance both drug release kinetics and dermal absorption, providing advantages for transdermal therapeutic applications [124]. A novel strategy leverages transport bioactive cargo such as miRNAs, mRNAs, and proteins, for overcoming the skin’s permeability barriers. Their incorporation into hydrogel formulations enhances drug encapsulation and facilitates targeted delivery, improving therapeutic efficacy in dermatological applications [1,125].

## 7. Preserving bioactivity of PDELNs in biocompatible hydrogels

**Figure 4.**
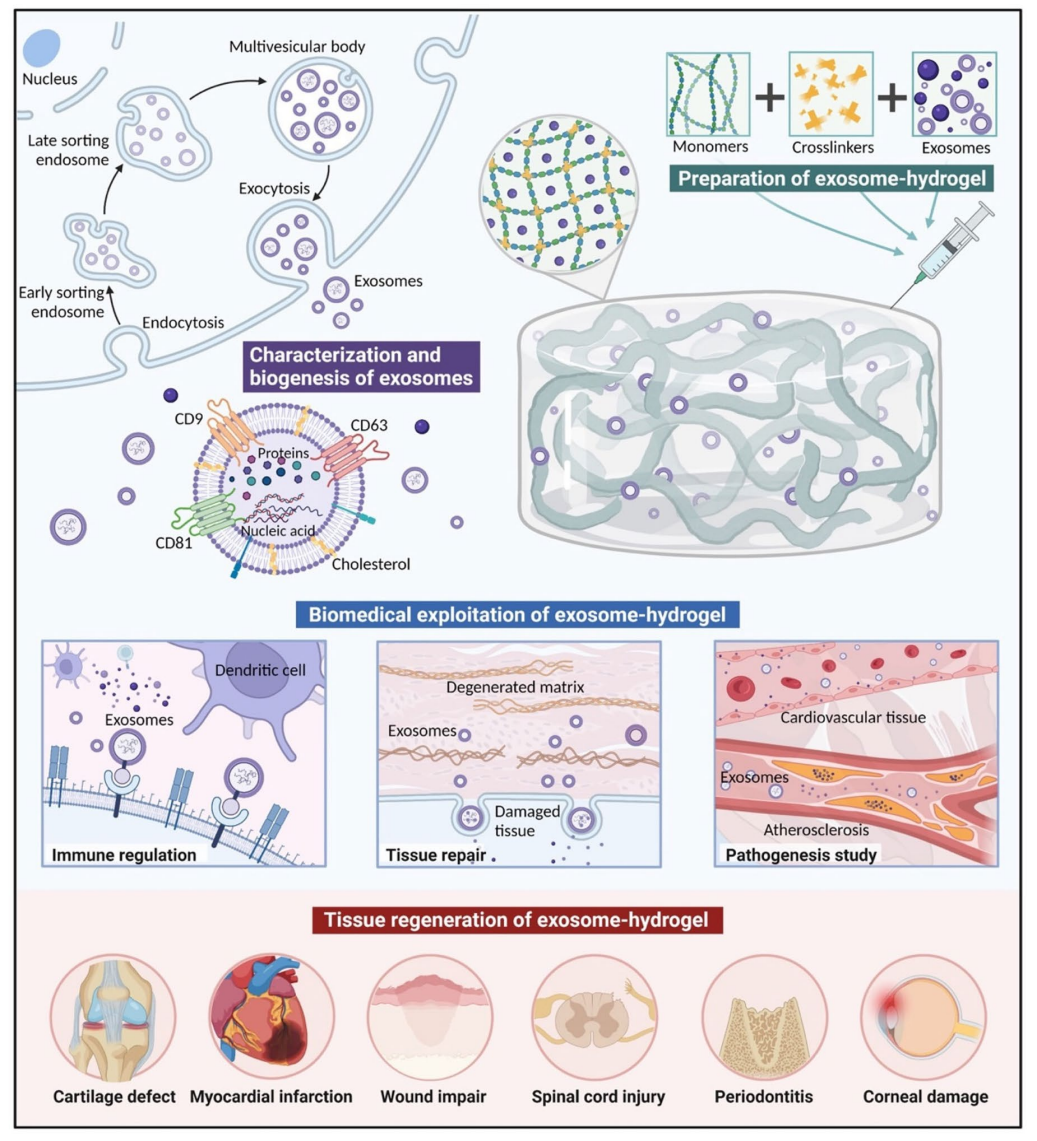
Illustration of biogenesis, characterization and biomedical uses of exosome-hydrogels. The upper left panel depicts formation of exosomes in the endosomal pathway which involves early and late sorting endosomes, multivesicular bodies, and exosome exo-cytosis. The upper right panel demonstrates the process of exosome-hydrogels preparation through combining exosomes with the monomers and crosslinkers to create a hydrogel matrix that is able to undergo long-term drug delivery. The main part shows biomedical applications of exosome-hydrogels, especially their functions as immune modulators, in tissue repair through matrix remodelling and regeneration of injured tissue in disease pathogenesis including atherosclerosis in cardiovascular tissue. The lower panel depicts the therapeutic nature of exosome-hydrogels in tissue regeneration therapy, such as cartilage defect repair, myocardial infarction, wound healing, spinal cord injury treatment, periodontitis treatment, and corneal damage repair. Adapted from reference [129] under a Creative Commons license (https://creativecommons.org/licenses/by/4.0/).

Various techniques have been established to embed PDELNs within hydrogels (Table 1), each balancing advantages and challenges related to preserving exosomal stability and biological functionality [126]. Schematic overview of exosome incorporation strategies in hydrogels depicts in Figure 4.

**Table 1.** Comparative summary of exosome–hydrogel incorporation strategies, highlighting retention capacity, release kinetics, and therapeutic outcomes.

Incorporation Technique	Retention in Hydrogel	Release Kinetics	Therapeutic Outcomes Reported	Ref
Direct Mixing	Low–moderate retention (exosomes may diffuse out rapidly due to weak physical entrapment). Stability may be affected by shear forces during mixing.	Often leads to initial burst release due to weak entrapment. No sustained release unless hydrogel mesh size is very small.	Useful when rapid local delivery is desired.	[127]
Electrostatic Binding	Moderate retention due to charge–based interactions between negatively charged exosomes and positively charged hydrogel polymers.	Displays medium-rate sustained release, slower than direct mixing. Release influenced by ionic strength and hydrogel composition.	Enhanced wound-healing and anti-inflammatory effects in some studies due to improved local residency.	[128]
Covalent Binding	High retention because of strong chemical linkages between exosome surface groups and hydrogel matrix.	Very slow release, often dependent on matrix degradation; may restrict exosome mobility.	Strong retention but possible loss of functional cargo (RNAs/proteins) due to chemical modification.	[128]
Encapsulation During Gelation / Crosslinking	High retention with uniform distribution throughout hydrogel network. Protects vesicles from degradation.	Demonstrates controlled, prolonged release	Most consistent therapeutic outcomes: enhanced skin repair, prolonged anti-inflammatory activity, improved angiogenesis, better tissue regeneration.	[129]
Degradation-Controlled Hydrogels (alginate–gelatin, enzymatically degradable systems)	Very high retention until hydrogel begins degrading; exosomes are gradually liberated as the scaffold breaks down.	Long-term controlled release depending on polymer degradation	Excellent for chronic wounds or prolonged therapy. Shows improved bioavailability and sustained skin-regeneration effects.	[130,131]

## 8. Challenges and future perspectives

Recent progress with PDELN-hydrogel systems highlights their therapeutic potential for skin diseases, yet challenges persist, particularly in large-scale production, maintaining stability, and enhancing skin penetration. Producing PDELNs at scale while preserving their bioactivity remains a major challenge [132,133]. Current isolation methods like differential ultracentrifugation and size-exclusion chromatography, are effective at laboratory scale but face major limitations in industrial applications, including low yield, high cost, and potential compromise of exosome bioactivity [134]. To overcome these limitations, emerging technologies like bioreactor systems, microfluidic platforms, and automated isolation processes are being investigated to improve scalability and enable consistent, high-quality exosome production (Table 2) [135]. Another major challenge is maintaining the stability of PDELNs within hydrogel formulations. As delicate vesicles, PDELNs require careful protection to preserve their bioactivity during both storage and application [136]. Traditional stabilization methods such as lyophilization, freeze-drying, and the use of cryoprotectants have been used to preserve exosome integrity. However, these approaches are still being refined, as further optimization is needed to ensure long-term retention of their therapeutic potential [137]. Future research aim to develop hydrogels with improved stabilization and investigate novel materials to protect PDELNs while maintaining release kinetics [21]. Effective therapeutic use of PDELNs is also challenged by limited ability to penetrate stratum corneum and reach deeper skin layers [138]. Strategies like microneedles, iontophoresis, and chemical penetration enhancers have been explored to improve transdermal delivery [54]. Future advancements in transdermal delivery systems, integrated with optimized exosome hydrogel systems, enhance bioavailability and therapeutic efficacy of PDELNs in wide range of skin disorders [139].

**Table 2.** Mechanistic insights from recent research to improve scalability and enable consistent, high-quality exosome production.

Aspect	Details	Key Findings	Ref
Nanotechnology in Hydrogel Modification	Use of nanotechnology for enhancing hydrogel encapsulation efficiency and controlled delivery of PDELNs.	Nanotechnology modifications adjusting pore sizes and surface charge, lead to improved exosome encapsulation, stability, and sustained release kinetics.	[140]
Hydrogel Structure Optimization	Modification of hydrogel pore size, surface characteristics, and mechanical properties to improve exosome stability and release profiles.	Pore size modulation and surface charge modifications ensure efficient exosome encapsulation, increasing both encapsulation capacity and release control.	[141]
Exosome Encapsulation Efficiency	Incorporation of nanomaterials, such as nanoparticles, into hydrogels to enhance exosome loading and release efficiency.	Nanomaterials improve the encapsulation of PDELNs, ensuring their bioactivity is preserved during the release phase for more targeted action.	[21]
Targeted Delivery *via* Surface Modification	Surface modification of PDELNs using ligands or peptides to enhance tissue-specific targeting in skin applications.	The attachment of targeting ligands or peptides onto exosome surfaces directs the PDELNs to specific skin cell receptors, enhancing the efficacy of treatments.	[18]
Tissue-Specific Targeting	Use of receptor-specific ligands or peptides on exosome surfaces for enhanced targeting of skin cells such as keratinocytes or dermal fibroblasts.	Targeted exosome delivery to specific skin layers (epidermis or dermis) reduces off-target effects and increases treatment precision for skin diseases.	[142]
Exosome-Targeted Therapy for Skin	Application of PDELNs-loaded hydrogel systems for targeted skin therapies, including wound healing and anti-inflammatory treatments.	Surface-modified PDELNs exhibit higher binding affinity for target cells, promoting faster wound healing and improved tissue regeneration.	[143]
Increased Bioactivity Preservation	Nanoparticles integrated into hydrogels help preserve the bioactivity of PDELNs during encapsulation, storage, and release.	The integration of nanoparticles helps protect PDELNs from degradation during the encapsulation process, preserving their therapeutic potential.	[144]

The regulatory framework governing clinical application of PDELNs formulations is evolving, with key concerns centred on their safety, potential toxicity, and immunogenicity. Comprehensive preclinical and clinical evaluations required to overcome these issues and establish standardized guidelines for their therapeutic use [145]. Since PDELNs are biologically derived nanovesicles, they would be regulated under existing biologics or advanced-therapy medicinal product (ATMP) frameworks rather than simple cosmetics or supplements. In particular, FDA and EMA typically require detailed characterization of source material, purity, potency, safety, and batch-to-batch consistency, and manufacturers must demonstrate the absence of contaminants or endotoxins while ensuring vesicle stability and identity. Good Manufacturing Practice (GMP) production presents major challenges for PDELNs, as scalable and reproducible isolation, purification, and quality control protocols are still underdeveloped, in contrast to mammalian-derived exosomes where standardized GMP pipelines exist. Lessons from mammalian exosome therapies highlight issues such as variability in vesicle composition, differences in isolation methods affecting potency, stability concerns during storage, and challenges in establishing reproducible functional assays, all of which are highly relevant for translating PDELNs into clinical applications. Furthermore, the potential immunogenicity and toxicity of PDELNs, particularly with long-term use or high doses, necessitate rigorous preclinical testing, as plant-derived vesicles may interact differently with the immune system compared to mammalian exosomes [128,136,146,147].

Another critical consideration is the long-term biocompatibility and biodegradability of exosome-hydrogel systems. Ensuring that these materials are safely metabolized or cleared from the body without eliciting adverse reactions is essential for their successful clinical translation and sustained therapeutic use [148]. Exosome formulations must preserve their stability and therapeutic efficacy throughout the treatment duration while also degrading safely *in vivo* to prevent toxic accumulation. Although hydrogels used for controlled exosome release are inherently biodegradable, it is crucial that their degradation byproducts are non-toxic and can be safely eliminated from the body [149].

Both the PDELNs and hydrogel matrices require comprehensive evaluation in preclinical models and clinical trials to ensure their degradation does not provoke chronic inflammation or other adverse effects. Looking forward, exploring novel PDELNs derived from medicinal plants known for their strong anti-inflammatory, antioxidant, or regenerative properties. Such PDELNs may offer enhanced therapeutic efficacy, especially in the wound healing, inflammatory skin disorders, and skin aging [150–152]. PDELNs are increasingly explored for their therapeutic potential in preclinical studies, clinical translation is still in its nascent stage. Currently, only few trials have been registered to investigate PDELNs in humans, reflecting the early exploration of their safety and efficacy. Among these, grape-derived exosomes (NCT01668849) are being evaluated for the treatment of radiation- and chemotherapy-induced oral mucositis. In this trial, patients receive daily oral doses over 35 days. Preclinical studies suggest that grape exosomes may facilitate tissue regeneration and reduce inflammation, but clinical results are not yet available, and trial remains active but not recruiting. Similarly, ginger- and aloe-derived exosomes (NCT03493984) are being studied for insulin resistance and chronic inflammation associated with polycystic ovary syndrome. These trials focus on evaluating safety, tolerability, and preliminary pharmacological effects in human subjects, though recruitment has not yet started [153]. Other PDELNs trials explore their potential as drug carriers. For instance, exosomes loaded with curcumin (NCT01294072) have been tested for colon cancer. Patients received oral doses for seven consecutive days, demonstrating the feasibility of using PDELNs for targeted drug delivery. Additionally, PDELNs are being investigated for metabolic and inflammatory conditions, including trials evaluating their effect on gastrointestinal health and systemic inflammation, although most of these studies are currently not recruiting or are in early-phase exploratory stages. Beyond plant-derived vesicles, clinical trials on mammalian exosomes provide valuable lessons for GMP production, vesicle characterization, and reproducible bioactivity assessment, which are directly relevant for future PDELN trials [153].

Several other registered studies illustrate the growing interest in exosomes as versatile therapeutic agents. Mesenchymal stem cell (MSC)-derived exosomes are being evaluated across indications such as bronchopulmonary dysplasia (NCT03857841), type 1 diabetes (NCT02138331), macular holes (NCT03437759), acute ischemic stroke (NCT03384433), and metastatic pancreatic cancer (NCT03608631). These trials, though mammalian in origin, provide critical insights into dosing strategies, administration routes (intravenous, subcutaneous, intradermal), purification methods, and safety monitoring that can guide the development of PDELNs therapies. Taken together, these studies highlight that while PDELNs is an emerging strategy due to their biocompatibility and natural origin, the clinical evidence remains limited, and future trials are needed to establish standardized protocols, confirm efficacy, and ensure safety in humans [153,154].

Hydrogel microneedle patch composed of GelMA tips loaded with mesenchymal stem cell (MSC)-derived exosomes, combined with backing layers containing antibacterial silver nanoparticles. Tested on full-thickness diabetic wounds, this system enhanced exosome delivery by creating microneedle-mediated microchannels, which improved penetration and resulted in accelerated wound closure and improved tissue regeneration [59,155]. Exosomes derived from *Rosa damascena*, when combined with microneedling, have demonstrated the ability to penetrate deeper dermal layers and improve scar texture and appearance in clinical settings. This approach leverages the temporary disruption of the stratum corneum by microneedling to enhance the delivery and efficacy of the PDELNs [86].

Detailed lipidomic, proteomic, and nucleic-acid profiling of vesicles from different plant sources should become standard practice this would allow establishment of “quality-control signatures” that ensure batch-to-batch reproducibility and safety, which is critical for regulatory approval. Evidence suggests that comprehensive molecular characterization is increasingly used in EV/vesicle research to improve translational reliability [156]. PDELNs hydrogel systems approval is subject to a number of regulatory challenges. In the U.S., exosome-based products are categorized as biologics and the FDA requires intense data on safety and efficacy, such as immunogenicity and potency tests. On the same note, EMA regulates these products as Advanced Therapy Medicinal Products (ATMPs), requiring adherence to Good Manufacturing Practice (GMP) and wide-ranging preclinical trials [157]. Exosome size, cargo, and bioactivity variability are also a major problem as they make it difficult to characterize and be consistent across batches [158]. Also, the aspects of drug-device of combination are also required in detail by regulatory authorities in case of hydrogels, which makes the approval route even more difficult. To handle these obstacles, standardized production guidelines, powerful potency assessments, and early regulatory interactions are all that is needed to make clinical translation successful.

## Methods

We conducted structured search of the literature on plant-derived exosomes and hydrogel-based drug-delivery systems for skin applications. Relevant studies were identified from PubMed, ScienceDirect, and Google Scholar using keywords including plant-derived exosomes, plant exosomes + skin, and plant exosomes + hydrogel. We selected Publications from 2010 to 2025 and focused on studies describing biological activity of plant-derived exosomes, as well as their therapeutic relevance for skin repair, regeneration, or wound healing. Research involving hydrogel formulations that incorporated plant-derived vesicles or comparable nano-carriers was also included. *In vitro*, *in vivo* (preclinical), and available clinical evidence were reviewed, while unrelated work (such as mammalian exosomes or non-skin studies) was excluded.

## Conclusion

PDELNs are emerging avenue in dermatology, providing biocompatible and low-immunogenic carriers for precise drug delivery. When combined with hydrogels, these systems improve drug stability, enable controlled release, and enhance skin penetration, making them effective for wound healing, inflammatory skin diseases, and tissue regeneration. However, moving these technologies from the lab to clinical practice requires addressing challenges in large-scale production, formulation stability, and regulatory approval. Future advancements may involve personalized therapies tailored to individual patients and conditions, as well as leveraging AI to optimize formulation design for greater efficacy and safety. Beyond medicine, these innovations have the potential to revolutionize the skincare and cosmeceutical industries, offering natural, smart, and customizable solutions for skin health and rejuvenation. By integrating traditional plant bioactives with cutting-edge nanotechnology and computational approaches, PDELNs hydrogel platforms are poised to transform both therapeutic and cosmetic dermatology.

## Data Availability

Data sharing is not applicable to this article as no new data were created or analyzed in this study.
